# Reducing the muscle activity of walking using a portable hip exoskeleton based on human-in-the-loop optimization

**DOI:** 10.3389/fbioe.2023.1006326

**Published:** 2023-05-04

**Authors:** Linghui Xu, Xiaoguang Liu, Yuting Chen, Linfan Yu, Zehao Yan, Canjun Yang, Congcong Zhou, Wei Yang

**Affiliations:** ^1^ Ningbo Innovation Center, Zhejiang University, Ningbo, China; ^2^ School of Mechanical Engineering, Zhejiang University, Hangzhou, China; ^3^ Spinal Cord Injury Rehabilitation Department, Ningbo Rehabilitation Hospital, Ningbo, China; ^4^ Hebei Heavy Machinery Fluid Power Transmission and Control Lab, Yanshan University, Qinhuangdao, China; ^5^ School of Medicine, Sir Run Run Shaw Hospital, Zhejiang University, Hangzhou, China

**Keywords:** muscle activity, portable hip exoskeleton, human-in-the-loop optimization, walking assist, surface electromyography (SEMG)

## Abstract

**Introduction:** Human-in-the-loop optimization has made great progress to improve the performance of wearable robotic devices and become an effective customized assistance strategy. However, a lengthy period (several hours) of continuous walking for iterative optimization for each individual makes it less practical, especially for disabled people, who may not endure this process.

**Methods:** In this paper, we provide a muscle-activity-based human-in-the-loop optimization strategy that can reduce the time spent on collecting biosignals during each iteration from around 120 s to 25 s. Both Bayesian and Covariance Matrix Adaptive Evolution Strategy (CMA-ES) optimization algorithms were adopted on a portable hip exoskeleton to generate optimal assist torque patterns, optimizing rectus femoris muscle activity. Four volunteers were recruited for exoskeleton-assisted walking trials.

**Results and Discussion:** As a result, using human-in-the-loop optimization led to muscle activity reduction of 33.56% and 41.81% at most when compared to walking without and with the hip exoskeleton, respectively. Furthermore, the results of human-in-the-loop optimization indicate that three out of four participants achieved superior outcomes compared to the predefined assistance patterns. Interestingly, during the optimization stage, the order of the two typical optimizers, i.e., Bayesian and CMA-ES, did not affect the optimization results. The results of the experiment have confirmed that the assistance pattern generated by muscle-activity-based human-in-the-loop strategy is superior to predefined assistance patterns, and this strategy can be achieved more rapidly than the one based on metabolic cost.

## 1 Introduction

For decades, scientists and engineers have been developing wearable robotic devices, such as exoskeletons, exosuits, and powered orthosis, to assist in rehabilitation or enhance human locomotion performance (Yang et al., 2016; Wei et al., 2020; Zhou et al., 2022). Recent progress in human-in-the-loop (HIL) optimization has demonstrated its great potential to improve wearable robotic devices’ performance (Ding et al., 2018; Koller et al., 2016; Zhang et al., 2017). Instead of using predefined assist torque profiles (Seo et al., 2016; Xue et al., 2019) or parameters-based functions (Young et al., 2017a; Lim et al., 2019; Yang et al., 2021), HIL optimization adopts the human’s physiological signals for the online iterative update of assist torque parameters. Consequently, wearable robotic devices are able to provide customized torque patterns to the subjects according to their biomechanical and physiological states (Young et al., 2017b; Wang et al., 2020).

In previous studies, metabolic expenditure was mostly used as the cost function for HIL optimization to generate customized torque patterns for both portable (Kim et al., 2019) and tethered (Lee et al., 2017; Zhang et al., 2017; Witte et al., 2020) exoskeletons. Despite the impressive metabolic expenditure reductions achieved, the research limitation is obvious. A long time is required to generate an optimized assistive torque profile. According to the study by Collins’s group, a 64-min walking period was required to optimize four control parameters based on metabolic expenditure (Zhang et al., 2017). Meanwhile, the mask for indirect calorimetry measurement was uncomfortable to wear. Therefore, different cost functions need to be explored. Zhang’s group successfully constructed a muscle-activity-based cost function for ankle-assistive torque pattern optimization. This proposed method could reduce the time by 50% compared to metabolic expenditure-based HIL optimization, since surface electromyography (sEMG) signals have less measurement time than metabolic expenditure (Han et al., 2021). At the same time, an sEMG sensor is a promising choice since it can sample the sEMG signals constantly and operates under less strenuous circumstances compared to metabolic cost measurement. Moreover, subjects adapt muscle activity more quickly than metabolic costs (Huang et al., 2012). Consequently, this muscle-activity-based optimization strategy is worth validating in other assistive exoskeletons, providing individual assistance for more joints.

Both Bayesian Optimization (BO) and Covariance Matrix Adaptive Evolution Strategy (CMA-ES) optimization algorithms have been verified in previous research and produced great results. BO utilizes the Bayesian technology to place a prior probability distribution over an unknown objective function and updates it to form the posterior probability distribution with the new function evaluation. CMA-ES employs an evolution strategy to generate a group of individuals in the first generation in a stochastic way and select parents for the next-generation based on their fitness (objective function value) to have better and better individuals. In a simulation environment, it has been theoretically demonstrated that BO, which updates objective function values based on probability distribution, has higher efficiency, while CMA-ES, which is based on stochastic search and evolution selection, has greater global search ability (Kim et al., 2019). In the previous study, CMA-ES optimized four parameters in at least 1 h (Zhang et al., 2017) with the ankle exoskeleton, and 22 parameters in 4 h with the hip-knee-ankle exoskeleton (Bryan et al., 2021) for metabolic expenditure optimization. BO optimized two parameters in 21.4 ± 1.0 min with the hip exoskeleton (Ding et al., 2018) for metabolic expenditure optimization. However, those two methods have not been compared in a human experiment under the same device with the same parameter set and optimization object. The answer would provide valuable instruction for researchers to conduct human experiments later.

The overall aim of this study is to provide a strategy for a portable hip exoskeleton that can determine the customized and optimal hip-assist torque patterns. A muscle-activity-based HIL optimization for a portable hip exoskeleton torque pattern generation was proposed. Then, a pilot test was first used to verify the feasibility of this framework. This was followed by tests of four volunteers to study the performance of both BO and CMA-ES for a hip exoskeleton. To our best knowledge, this is the first study of a portable hip exoskeleton torque generation with a muscle-activity-based cost function for HIL optimization. The results of this work are beneficial for guiding the assistive and resistive torque/force profiles’ optimization of upper/lower limb wearable devices for augmentation and physical training, respectively. Overall, the contributions of this study are listed as follows: 1) the HIL optimization strategy based on sEMG signals is proposed to lower the time investment of each iteration and 2) the effectiveness of both BO and CMA-ES algorithms was validated and compared through experimental results.

## 2 Materials and methods

### 2.1 Portable hip exoskeleton

**FIGURE 1:**
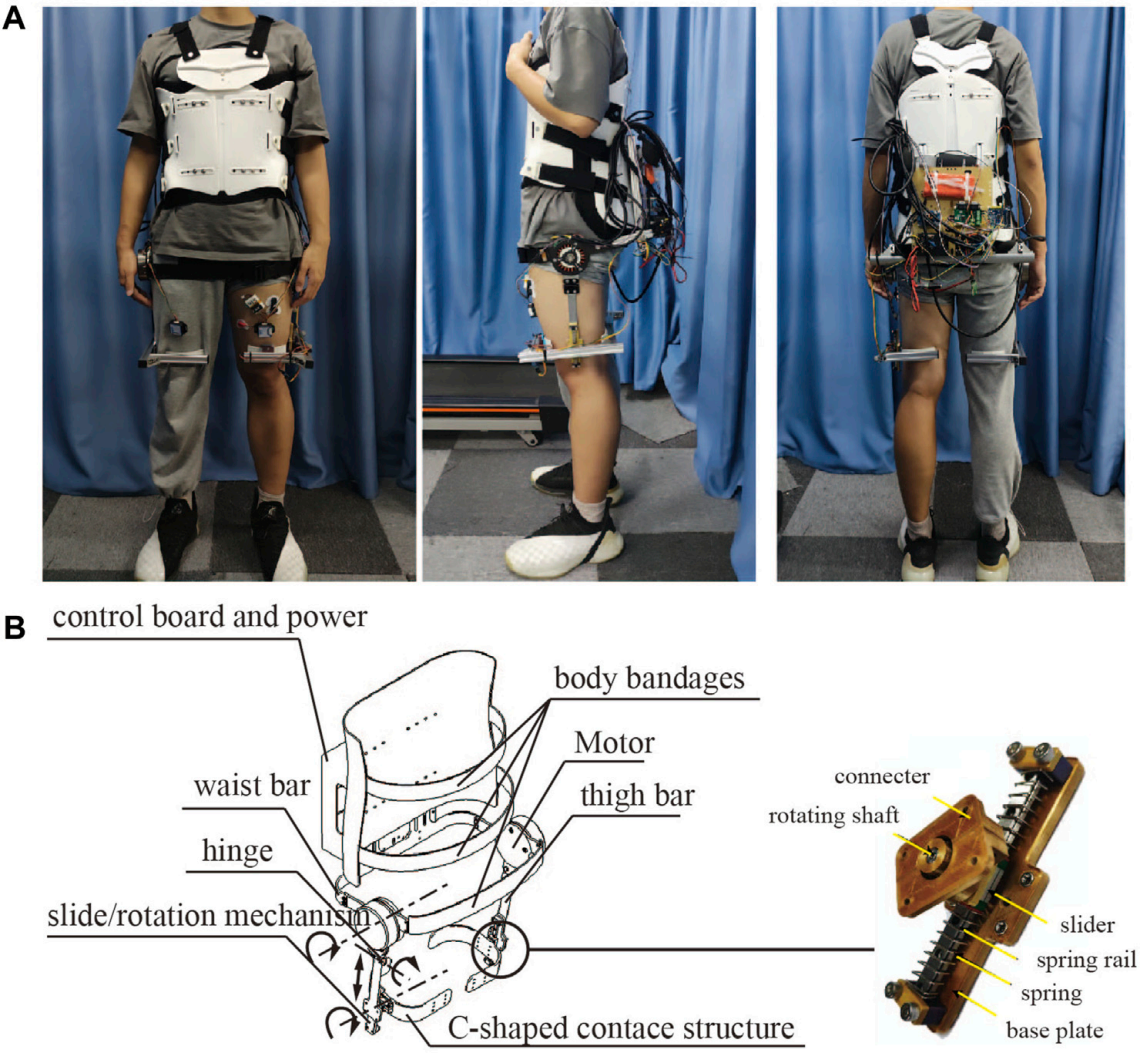
Portable hip exoskeleton. **(A)** The front, side, and rear view of a subject wearing the exoskeleton. **(B)** Exoskeleton structure.

The portable hip exoskeleton was developed initially in our previous study (Yang et al., 2021; Yu et al., 2022) and received structural optimization for better joint alignment in this study. Figure 1 shows the structure of the portable hip exoskeleton. Both left and right hip joint mechanisms have one active and three passive degrees of freedom (DoF). The active DoF for hip flexion and extension is driven by a motor module (JA90 motor and DS300 motor driver, RoboCT Co., Ltd., Hangzhou, China). Then, a damping hinge with a moment damping of 2.3 Nm on the upper part of the leg bar provides a passive DoF. Its functionality for wearing the portable hip exoskeleton easily and reducing the unexpected human-exoskeleton interaction force during walking has been shown in the experiment, although it is not aligned with the center of human hip joint adduction and abduction. The other two passive DoFs are introduced by the slide/rotation mechanism (Figure 1B) which connects the C-shaped contact structure and thigh bar. With these two passive DoFs, the C-shaped contact structure will passively slide along the spring rail and rotate on the rotating shaft with wearer ambulation, which will eliminate unexpected interaction forces here and improve efficiency as well as comfort in transmitting the assistive torque. Spring can help the slider recover the original position. For adaptation to different human body types, the waist and thigh bars can be extended and shortened thanks to the lockable sliders. Body bandages are utilized to secure the upper body of the wearer and the exoskeleton, particularly the motor and human-exoskeleton interface parts, during ambulation to ensure that the positions of these interface parts and motor shafts are maintained. Two IMUs are arranged near the fixed straps of the thighs on both sides, which are used to measure the hip flexion and extension angles, and angular velocities. During assistive walking control, the microprocessor unit (MCU) reads the IMU information through the serial port, and after processing by the human locomotion phase estimation algorithm, it can identify the gait phase and generate the assistive torque, then control the motor driver by PWM. The control board realizes wireless data transmission with the host computer through Bluetooth for real-time monitoring of the status of the control system. The overall structure of the lower limb hip exoskeleton is mainly composed of aluminum alloy, carbon fiber, and plastic, with a total weight of 3.5 kg. Each motor power is 200 W and can provide 17.8 N m constant torque and 37.8 N m peak torque.

### 2.2 Muscle activity

A large number of muscles in the lower limbs are involved during walking and those muscles are controlled by the bioelectrical activity of spinal motor neurons under the control of the cerebral motor cortex, which leads to a faint electric signal on the skin’s surface. Those faint electric signals are sEMG signals and their root-mean-square (RMS) values can reflect the strength of the electromyographic signal, which essentially represents the degree of muscle activity (Han et al., 2021). Consequently, in this study muscle activity is calculated as follows.

$$I_{muscle}=\sqrt{\frac{1}{n}\sum_{i=1}^{n}\left(S_{i}^{2}\right)},$$ (1)

where $I_{muscle}$ represents the muscle activity and S is the normalized sEMG value collected at time serial number i .

The measurement of sEMG signals is performed in a non-invasive way by a single or a group of electrodes placed on the skin surface of the muscle to be measured (Zhou et al., 2021). A wireless sEMG sensor with a compact size and sampling frequency of up to 2,000 Hz was used in this study. During the experiment, the sEMG signals of the rectus femoris (RF) muscle were acquired as the HIL optimization objective. We have chosen the rectus femoris muscle for our experiment for a few reasons. Firstly, it plays a vital role in hip flexion during walking. Secondly, it can be easily measured with an EMG sensor without interfering with the exoskeleton hardware. To get online muscle activity for the online HIL optimization test, the raw sEMG signals are first processed through a fourth-order 20–200 Hz band-pass Butterworth filter, full wave rectifier, and 10 Hz low-pass filter, and normalized by maximum sEMG signal value, which is determined through finding the maximum EMG value during te hacclimation protocol of 1-min walking without the assistance of the exoskeleton (more detail is provided in 2.6.1 Acclimation before the experiment). Then, a series of 20-s time windows (each time window size contains about 10 gait cycles) is used to segment the sEMG data flow online with a 0.5 s step. Finally, in each window, the muscle activity is acquired within each time window and averaged (Lariviere et al., 2008).

### 2.3 Human locomotion phase

The human locomotion phase is estimated through a phase oscillator as it is a simple and efficient method that relies only on the angle and angular velocity (De la Fuente et al., 2020). The human locomotion phase φ estimated from the phase oscillator can be expressed as:

$$\varphi =\mathrm{atan}2\left(\dot{\theta },\theta \right),$$ (2)

where θ and $\dot{\theta }$ are the current joint angle and angular velocity, respectively. To improve the performance of the algorithm, adaptive phase translation and scaling strategies are applied.

$$\left\{\begin{array}{c} \hat{\theta }\left(t\right)=s\left(t\right)\times \theta \left(t\right)+\alpha \left(t\right)\\ \hat{\dot{\theta }}\left(t\right)=\dot{\theta }\left(t\right)+\beta \left(t\right) \end{array}\right.,$$ (3)

where $s\left(t\right)$ is the scaling parameter and $\alpha \left(t\right)$ and $\beta \left(t\right)$ are the translation parameters for hip angle and angular velocity, respectively (Hong et al., 2021).

### 2.4 Assistive torque profile

**FIGURE 2:**
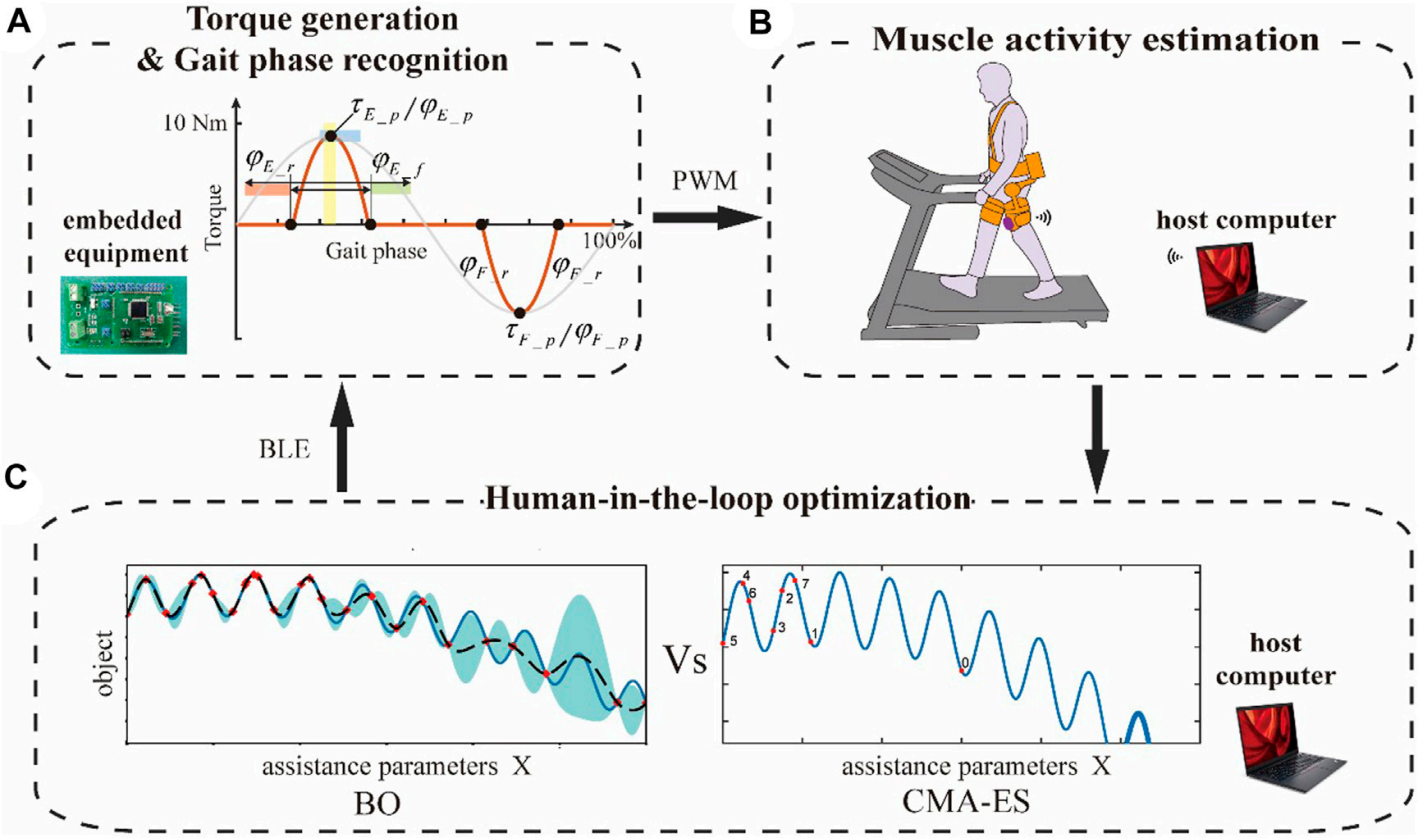
Human-in-the-loop optimization experiment setup. **(A)** Assist torque profile parameterization and diagram of the range of each parameter. **(B)** Muscle activity estimation. The purple part represents sEMG sensors, the orange part portable hip exoskeleton, and the grey part treadmill. **(C)** Human-in-the-loop optimization for the next better assist torque profile parameter set.

To employ the HIL optimization for exoskeleton assistance, the parameterized control scheme is required for the portable hip exoskeleton. We defined an assist torque profile inspired by the biological hip joint torque, which is constructed as a piece-wise combination of five curves (Figure 2A). Except for the zero-constant curves, all others are quadratic functions connected smoothly. The whole assist torque profile can be defined totally by rising time, fall time, peak time, and peak torque of extension and flexion torque.

To prevent obvious non-optimal solutions in the process of optimization and accelerate the convergence speed, the parameter set range of the assist torque profile is artificially limited as follows.

$$\left\{\begin{array}{c} \tau_{E\_p}\in \left[0Nm,10Nm\right]\\ \begin{array}{c} \varphi_{E\_p}\in \left[20\%,30\%\right]\\ \varphi_{E\_r}\in \left[10\%,20\%\right]\\ \varphi_{E\_f}\in \left[10\%,20\%\right] \end{array}\\ \begin{array}{c} \tau_{F\_p}=\tau_{E\_p}\\ \varphi_{F\_p}=50\%+\varphi_{E\_p}\\ \begin{array}{c} \varphi_{F\_r}=\varphi_{E\_r}\\ \varphi_{F\_f}=\varphi_{E\_f} \end{array} \end{array} \end{array}\right..$$ (4)

A predefined torque profile is used to compare to the parameterized torque profile (De la Fuente et al., 2020; Yang et al., 2021).

$$\tau \left(t\right)=k∙\sin\left(\varphi \right),$$ (5)

where k is the assistive factor and φ the human locomotion phase. According to our previous experiments, k=4.5 shows a good assistance effect. Thus, this is used to compare with HIL optimized torque profile in the verification test (Verification experiment section).

### 2.5 Human-in-the-loop experiment setup

Bayesian optimization (BO) and CMA-ES were used in our HIL optimization experiment setup as optimizers, which are both suitable for complex human musculoskeletal model optimization and have been verified in previous studies.

Both optimization methods had initialization and optimization phases during implementation. BO was initialized by estimating muscle activity for one iteration with a pseudo-randomly chosen assist torque profile parameter set. During the optimization phase, BO used the Gaussian process with Matern kernel to build the posterior distribution of the human model-based collected dataset, and we used the expected improvement utility function to find the next optimal. The parameter set was defined in Section 2.4. The key initial parameters’ values are listed in Table 1, which is set according to previous studies and simulations (Zhang et al., 2017; Ding et al., 2018; Kim et al., 2019). CMA-ES was initialized the same way as BO, and the initial sampling step size, covariance matrix, and mean value are fixed. In the optimization phase, during each generation, CMA-ES sampled assistive torque profile parameter set λ times and chose the first to $N_{best}$ -th best parameter sets, based on their muscle activity results. Those best parameter sets were used to update the covariance matrix, mean value, and sampling step size for the next-generation. Related values of the key initial parameters are listed in Table 2, which is set according to the literature (Hansen, 2016; Zhang et al., 2017).

**TABLE 1:** Bayesian optimization parameters list in HIL optimization.

Parameter	Value	Meaning
$\tau_{E\_p}$	(0 N m, 10 N m)	Peak torque range
$\varphi_{E\_p}$	(20%, 30%)	Peak time range
$\varphi_{E\_r}$	(10%, 20%)	Rise time range
$\varphi_{E\_f}$	(10%, 20%)	Fall time range
*N*	1	Initial iteration number before optimization
**x**_**0**_	pseudo-randomly chosen	Initial assistive torque profile parameter set value
l	1.0	Matern kernel width coefficient
υ	2.5	Matern kernel smooth coefficient
ξ	0.1	Trade-off coefficient in the expected improvement utility function. A larger trade-off coefficient gives Bayesian optimization a stronger exploitation ability.

**TABLE 2:** CMA-ES optimization parameters list in HIL optimization.

Parameter	Value	Meaning
$\tau_{E\_p}$	(0 N m, 10 N m)	Peak torque range
$\varphi_{E\_p}$	(20%, 30%)	Peak time range
$\varphi_{E\_r}$	(10%, 20%)	Rise time range
$\varphi_{E\_f}$	(10%, 20%)	Fall time range
**x**_**0**_	(1.36 N m, 25%, 15%, 15%)	Initial sampling mean value
$\sigma_{0}$	1.400	Initial sampling step size.
C	Identity matrix	Initial sampling covariance matrix
λ	8	Population size
$N_{best}$	4	The number of selected points
*W*	(0.53, 0.29, 0.14, 0.04)	Positive weight coefficients for recombination
$\mu_{eff}$	2.600	Variance effective selection mass for the mean
$c_{\mu }$	0.051	The learning rate for the rank-µ update of the covariance matrix update
$c_{1}$	0.065	The learning rate for the rank-one update of the covariance matrix update
$c_{c}$	0.500	The learning rate for cumulation for the rank-one update of the covariance matrix
$c_{\sigma }$	0.400	The learning rate for the cumulation of the step-size control
$d_{\sigma }$	1.400	Damping parameter for step-size update

The whole experiment setup is shown in Figure 2. Participants walk on the treadmill wearing a portable hip exoskeleton with sEMG sensors placed on the RF muscle of the left leg, detection electrodes adhered to the RF muscle of the left leg, and reference electrodes adhered to the adipose area of the medial side of the left leg. The walking speed is decided according to a preliminary experiment based on comfort and safety. During the experiment, the control board of the portable hip exoskeleton generates assistive torque online based on the human locomotion phase estimation method and parameterized torque profile (Figure 2A). Then, the control board can receive the latest assistive torque parameter set from a host computer and control the motor to provide target assistive torque by pulse width modulation (PWM). A participant wearing the portable hip exoskeleton and sEMG sensor receives the assistive torque. The host computer acquires the sEMG of RF muscle in real-time through WIFI and extracts the muscle activity characteristic (Figure 2B). BO and CMA-ES are executed in Python on the host computer, generating the next potential optimal assist torque profile parameter set, which is then sent to the control board of the exoskeleton (as shown in Figure 2C) to close the loop.

### 2.6 Human-in-the-loop experiment protocol

**FIGURE 3:**
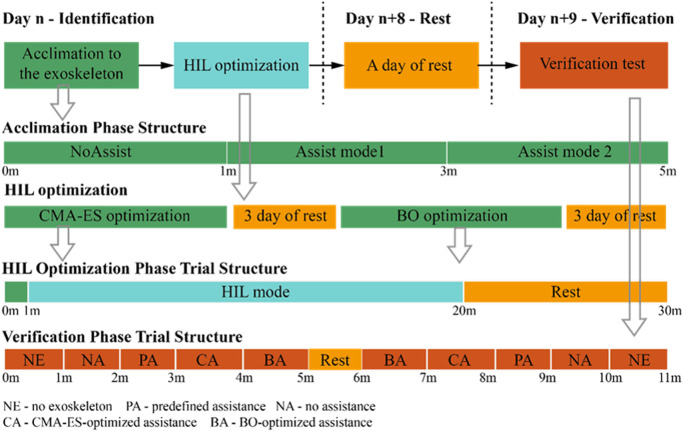
Human-in-the-loop experiment protocol flowchart. In the experiment, CMA-ES and BO optimization protocols are separated into two periods in case of muscle fatigue. CMA-ES optimization is first conducted and then BO optimization is conducted after 3 days.

The HIL optimization experiment consists of two sessions. One experienced male volunteer (weight 49.5 kg, height 1.61 m, age 22 years), who had attended previous studies on the exoskeleton of this type, was involved in the first session; its protocol is shown in Figure 3. According to the volunteer’s preference, the treadmill speed was set at 3 km/h and the assist torque amplitude was set within 0.0∼6.8 Nm. The goal of this session was to investigate the efficiency of sEMG-based HIL optimization and compare its optimal assistive torque profile with a predefined one. For the second session, four volunteers (weight 67.1 ± 10.2 kg, height 1.72 ± 0.09 m, age 25.8 ± 4.8 years) including the one who attended the first session were recruited. In this session, the treadmill speed was set at 3.6 km/h and the assist torque amplitude was set within 3.0∼10.0 Nm. The protocol is similar to the first session. The volunteers were randomly divided into two groups (two volunteers for each group). The first group conducted Bayesian-based HIL optimization first followed by CMA-ES-based HIL optimization. The second group conducted CMA-ES-based HIL optimization first followed by Bayesian-based HIL optimization. The goal of this session was to investigate the influence of the order of the two typical optimizers on the muscle activity reduction results. Informed consent was obtained from each volunteer, which was approved by the ethics committee of College of Biomedical Engineering&Instrument Science, Zhejiang University.

#### 2.6.1 Acclimation before the experiment

Before formal experiments, each participant had 5 min to adapt to wearing and coordinating with the portable hip exoskeleton. Thus, the participants gradually adapted to the assistance experience provided by the exoskeleton and did not intentionally resist the assistance torque. The acclimation protocol consisted of 1-min walking with no-assistance-mode exoskeleton step followed by two 2-min walks with predefined maximum and minimum peak torque assistance profiles. Stable walking was observed during the whole procedure to make sure the subject adapted to the device.

#### 2.6.2 Human-in-the-loop optimization experiment

For the first session, CMA-ES optimization was first conducted, and then BO optimization was conducted after 3 days. For the second session, BO optimization was first conducted for the first group followed by CMA-ES optimization, and the order was exchanged for the second group. Each HIL optimization was completed in 1 day. In each HIL optimization phase trial, the participant walked with the portable hip exoskeleton and started running the HIL optimization algorithm after walking was stable in the beginning 1-min walking in no-assistance mode. We required each participant to walk steadily for 25 s after adaptation to assistive torque change in each HIL optimization iteration. During steady walking, RF muscle activity was calculated online by the collected sEMG signal. By marking the location of the landing points on the treadmill, the participant was required to step on the points to control the stride length during walking. In addition, prolonged walking tends to cause muscle fatigue, so the participant rested for 10 min after 20 min of walking. During the rest time, we would check the portable hip exoskeleton hardware to ensure that the next round of experiments would perform properly.

The HIL optimization process ended when the optimization algorithm results converged. The CMA-ES optimization process is considered converged when the sampling step size change and normalized muscle activity change are below the termination threshold for five continuous iterations. And BO optimization process is considered converged when the iteration-to-iteration normalized muscle activity change is below the termination threshold for five continuous iterations.

#### 2.6.3 Verification experiment

After a whole day’s rest, a validation experiment was conducted to verify the assistive effect of the optimal assistive profile obtained from the HIL optimization experiment. For both sessions, each participant was asked to walk at the same speed as the HIL optimization experiment wearing the portable hip exoskeleton with an sEMG sensor arranged at the same position as the HIL optimization experiment. Each participant was asked to walk under multiple conditions, namely, no exoskeleton (NE), predefined assistance (PA), no assistance (NA), CMA-ES-optimized assistance (CA), and Bayesian-optimized assistance (BA), as shown in Figure 3, for 1 min. To minimize the effects of experiment order, adaptation, and fatigue, the participant experienced the same assistance pattern twice, but in the opposite order after 1-min rest. Two experimental results under the same conditions were averaged.

## 3 Results

### 3.1 Controller results

**FIGURE 4:**
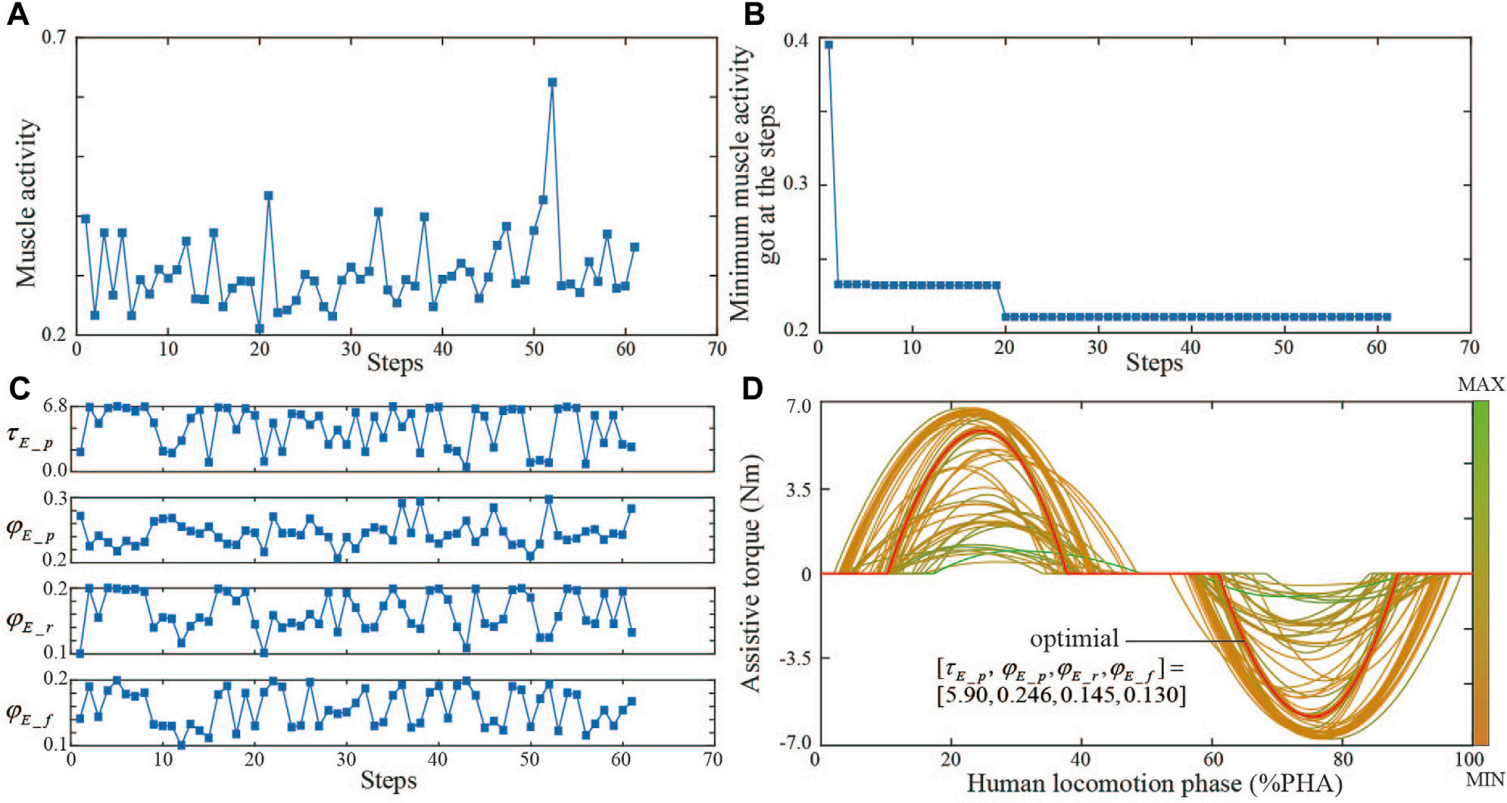
Bayesian optimization iteration trial. **(A)** Muscle activity during each optimization step. **(B)** Optimal muscle activity got at the current step. **(C)** Optimal parameter set of assist torque profile during each iteration. **(D)** Selected assist torque profiles during each optimization step.

**FIGURE 5:**
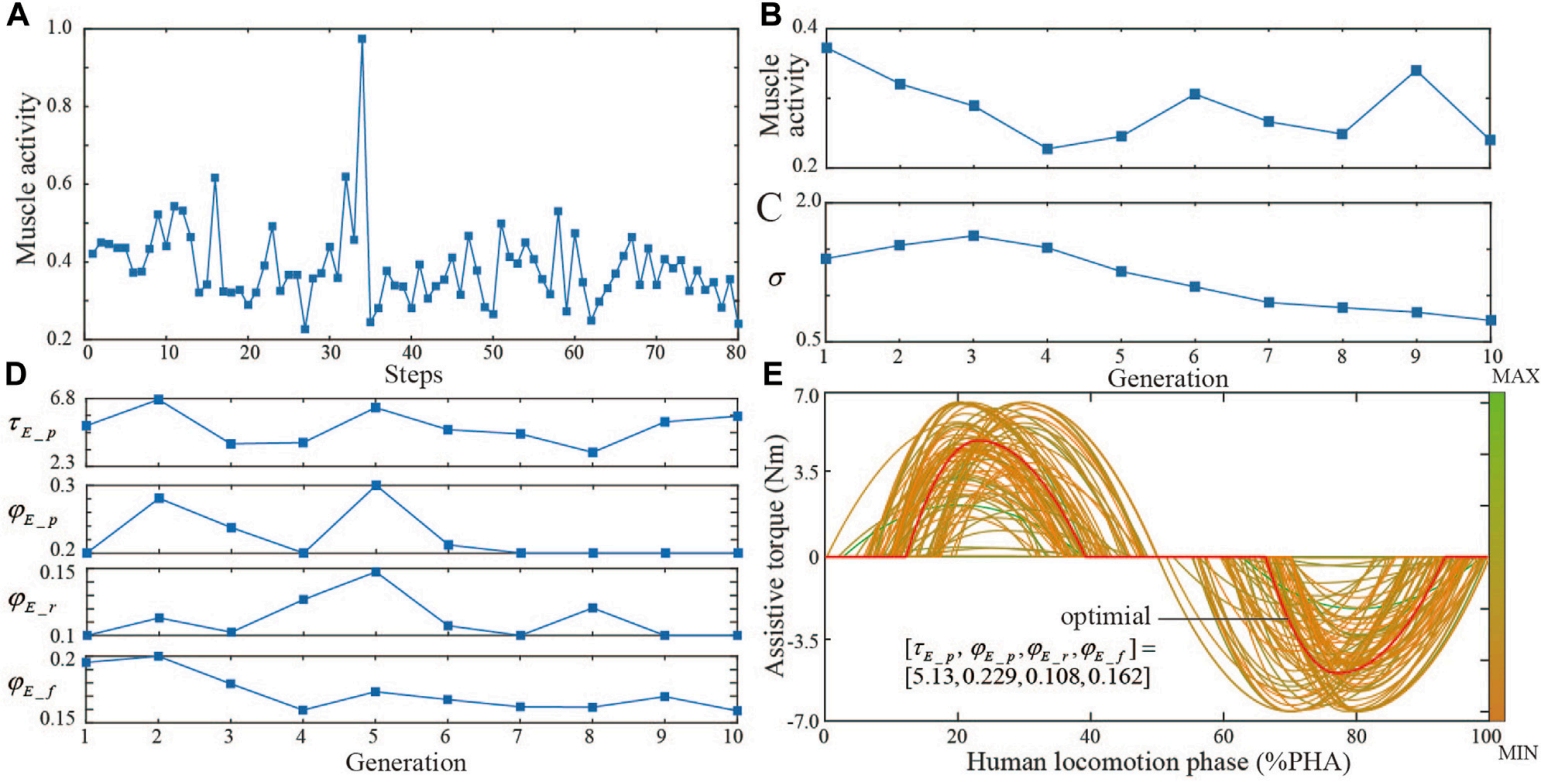
CMA-ES iteration trial. **(A)** Muscle activity during each optimization step. **(B)** Optimal muscle activity during each generation. **(C)** Step size during each generation. **(D)** Optimal parameter set of assist torque profile during each generation. **(E)** Selected assist torque profiles during each optimization step.

For the first session, the BO iteration process and results are shown in Figure 4. The whole optimization includes 61 iteration steps and takes about 1 hour. Muscle activity and assist torque profile parameters at every iteration step show the exploration-exploitation search strategy path (Figures 4A, C). The optimal objective is reached at the 20th iteration step and has no change in the last 40 iteration steps (Figure 4B). The optimal assistive torque profile parameter set is ${\left[\tau \right.}_{E\_p},\varphi_{E\_p},\varphi_{E\_r},\varphi_{E\_f}]=$ [5.90, 0.246, 0.145, 0.130]. Contrary to intuition, the optimal assist torque profile does not necessarily always have the largest peak torque, and a peak torque that is too low or too high is more likely to result in increased human effort during walking. The CMA-ES iteration process and results are shown in Figure 5. The whole optimization includes 10 generations (80 iteration steps) and takes about 1.5 h. Muscle activity and assist torque profile parameters at every iteration step show the CMA-ES’ search strategy path (Figures 5A, D). Optimal muscle activity and assist torque profile parameters gradually converge with iteration (Figures 5B–D). The optimal parameter set is ${\left[\tau \right.}_{E\_p},\varphi_{E\_p},\varphi_{E\_r},\varphi_{E\_f}]=$ [5.13, 0.229, 0.108, 0.162]. Among all the assistive torque profiles selected during the CMA-ES, results are most similar to BO’s (Figure 5E).

**FIGURE 6:**
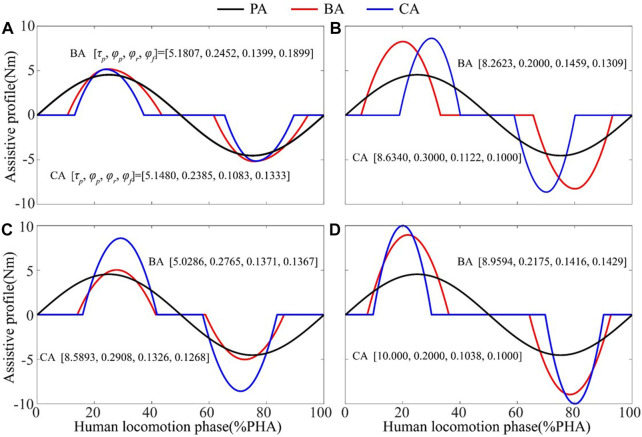
Optimized torque profiles based on Bayesian and CMA-ES algorithms during the second session. **(A)** Subject 1. **(B)** Subject 2. **(C)** Subject 3. **(D)** Subject 4.

For the second session, the optimized torque profiles based on Bayesian and CMA-ES algorithms for each participant were shown in Figure 6. For each participant, the optimized torque profile parameter sets vary between two optimizers, and the corresponding torque profiles are different in both torque amplitude and timing.

### 3.2 Biomechanical results

**FIGURE 7:**
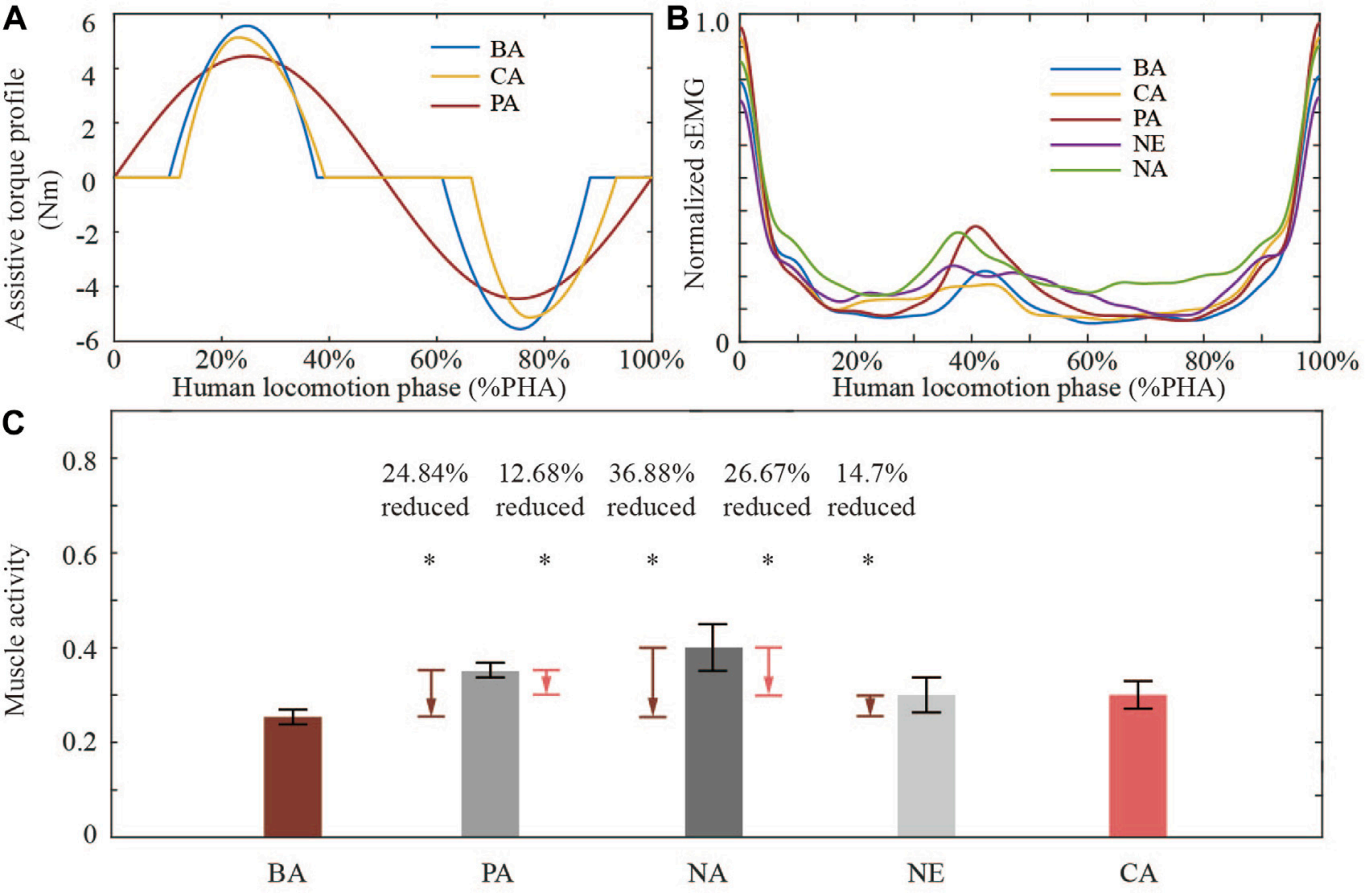
HIL optimized assist torque profile verification experiment results. **(A)** CMA-ES-optimized, BO-optimized, and predefined assistive torque profiles. **(B)** Averaged normalized sEMG signal in a gait period during the verification experiment. **(C)** Muscle activity results in five experimental conditions. BA and CA are compared with other conditions utilizing the *t*-test. Star “*” symbolizes that there is a significant difference between each other (*p* << 0.05, statistical power > 0.9).

Assist torque profiles used in the verification experiment for the first session are shown in Figure 7A. We averaged the RF sEMG signal and generated the mean single-period sEMG curve under each condition (Figure 7B). The mean sEMG value under NE is lower than that under NA in almost all gait phases, and that under BO has a lower value in most gait phases compared with that under NE condition. Quantitative muscle activity results (Figure 7C) show that BO optimized assistive torque profile can reduce about 24.84%, 36.88%, and 26.67% RF muscle activity compared with that under PA, NA, and NE conditions, respectively. CMA-ES-optimized assistive torque profile can reduce about 12.68% and 26.67% compared with that under PA and NA conditions, respectively. The above results have a significant statistical difference (*p* << 0.05) under the independent sample *t*-test. And results show there is no significant difference between CA condition and NE condition.

**FIGURE 8:**
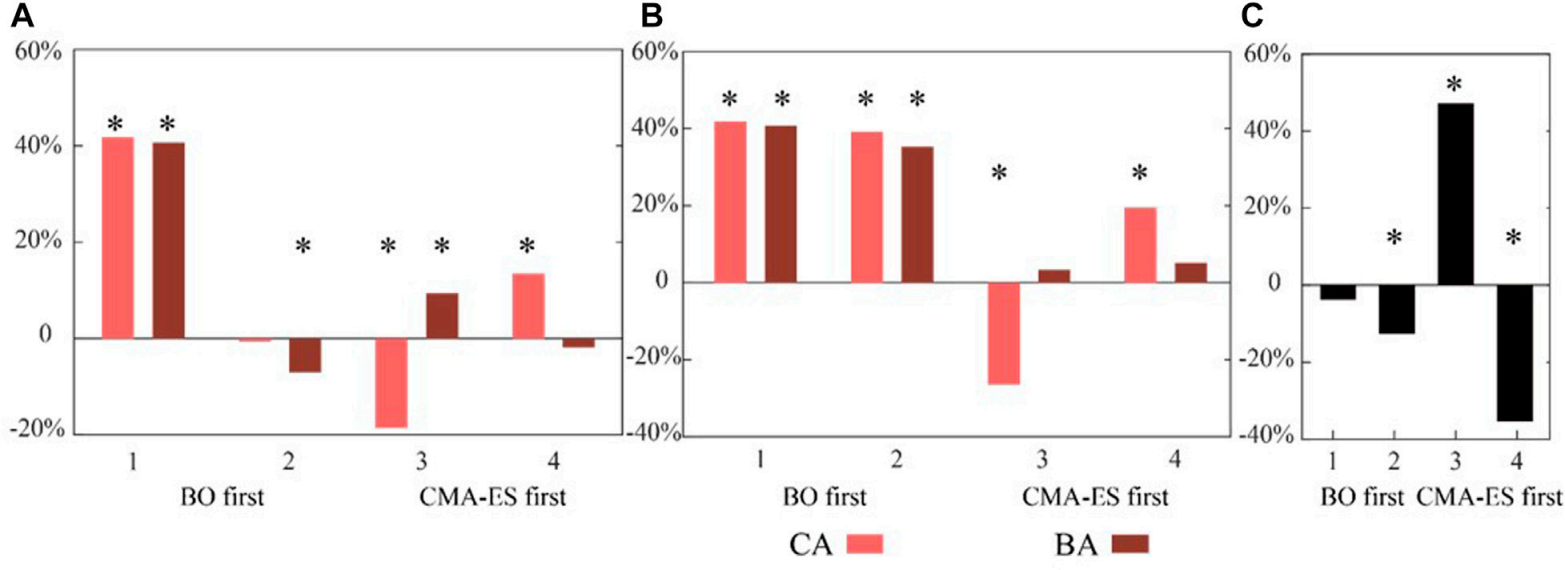
Quantitative muscle activity results of HIL-optimized assistive torque profile verification experiment for each participant. The *X*-axis label represents four subjects involved in the second session of experiments. The symbol “*” indicates a statistically significant difference (*p* << 0.05, statistical power > 0.9). **(A)** Muscle activity reduction in CA and BA compared with PA. **(B)** Muscle activity reduction in CA and BA results compared with NA. **(C)** Muscle activity reduction in CA compared with BA.

Quantitative muscle activity results of the HIL-optimized assistive torque profile verification experiment for each participant in the second session were shown in Figure 8. Subject 1 and Subject 2 belong to the first group that conducted BO first, and Subject 3 and Subject 4 belong to the second group that conducted CMA-ES optimization first. In the first group, no significant difference in muscle activity is found between BA and CA for participant 1, and a slight reduction of CA compared to BA is found for participant 2. In the second group, there is a significant reduction of muscle activity during BA compared to that of CA for Subject 3. However, for Subject 4, there is a significant increase in muscle activity during BA compared to that of CA. Consequently, changing the order of the optimizers during HIL optimization does not obviously affect the performance of both optimizers. The muscle activity of each assistance mode and the muscle activity reduction percentage of CA and BA relative to PA, NE, and NA are listed in Table 3. A negative number stands for a muscle activity increase of CA or BA compared to PA, NE, or NA. For each participant, the maximum reduction percentage of CA and BA is in bold.

**TABLE 3:** Muscle activity results for each subject during each assistance mode.

Subject No.	HIL optimizer	PA	NE	NA	CA	BA
1	Muscle Activity (μV)	143.97	114.20	130.41	75.88	77.35
	CA reduction (%)	**47.29**	33.56	41.81	—	—
	BA reduction (%)	**46.27**	32.27	40.69	—	—
2	Muscle Activity (μV)	66.68	60.14	110.33	67.09	71.36
	CA reduction (%)	−0.61	−11.56	**39.19**	**—**	—
	BA reduction (%)	−7.02	−18.66	**35.32**	**—**	—
3	Muscle Activity (μV)	106.66	66.93	100.00	126.52	96.69
	CA reduction (%)	**−18.62**	−89.03	−26.52	—	—
	BA reduction (%)	**9.35**	−44.46	3.31	—	—
4	Muscle Activity (μV)	61.29	51.53	65.84	53.05	62.45
	CA reduction (%)	13.44	−2.95	**19.43**	**—**	—
	BA reduction (%)	−1.89	−21.19	**5.15**	**—**	—

## 4 Discussion

The primary goal of this study was to validate an sEMG-based hip exoskeleton control scheme that automatically tunes the assist torque patterns with the wearer’s lower limb muscle activities through HIL optimization. To the best of the authors’ knowledge, it is the first time that muscle activity has been adopted for HIL optimization for a portable hip exoskeleton.

### 4.1 Comparison of torque generation algorithms

The results of the first session indicate that there are some advantages for sEMG-based HIL optimization control for controlling robotic hip exoskeletons compared to the predefined torque profile control. Although the predefined torque profile control can reduce muscle activity (16.02% reduction, *p* << 0.05) compared to walking during assist-off mode (the hip exoskeleton provides no assist torque), the HIL optimization had a larger RF muscle activity reduction than the predefined one (Figure 7). The HIL optimization with BO could reduce the RF muscle activity by 24.84% and 14.7% (*p* << 0.05) compared to predefined torque profile control and walking without the hip exoskeleton, respectively. The HIL optimization with CMA-ES could reduce the RF muscle activity by 12.68% (*p* << 0.05) compared to predefined torque profile control. However, when compared to walking without the hip exoskeleton, there was no significant difference. A possible explanation for this result was that the relatively long HIL optimization walking test may lead to the subject sweating which could cause signal drift of the target sEMG, leading to the non-optimized torque pattern. There is a significant difference between BO and CMA-ES optimization results. The result of BO is much better than that of CMA-ES by 13.93%. It turned out that CMA-ES was stuck into a local minimum. This may be influenced by the initial individual group selected pseudo-randomly and the random offspring selection. In the experiment, BO shows its great global optimization efficiency and found the optimal assistive torque parameter set in 20 steps. However, from the selected assistive torque profiles during each optimization step, CMA-ES searched a larger range of parameter sets than BO (Figures 4, 5). Less efficiency of CMA-ES did not transfer the better global search into faster optimization speed and better optimization results. Another possibility might be that the participant in the first session conducted CMA-ES-based HIL optimization, and then the Bayesian-based HIL optimization. The participant may have become gradually more familiar with exoskeleton-assisted walking during the first optimization trial, leading to better interaction and performance in the later optimization trial. To eliminate the influence of the order of both optimizers, we divided four volunteers into two groups randomly, and exchanged the order for two groups in the second session.

The muscle activity results of the second session indicate that the order exchange of two optimizers does not obviously affect the HIL optimization performance of both optimizers. According to the verification experiment results of four participants, both BO and CMA-ES are capable of generating assist torque profiles for muscle activity reduction with similar percentages, except for Subject 3. Meanwhile, only Subject 1 shows muscle activity reduction of CA and BA when compared to NE. One explaination may be that only Subject 1 is experienced with exoskeleton-assisted walking. During HIL optimization, the other three participants were prone to adjusting their stride length or stride frequency when new torque profiles were generated and applied. This may influence the convergence of HIL optimization.

According to the results of torque pattern optimization tests, HIL optimization with both BO and CMA-ES algorithms can be converged within a limited time. Besides the similar muscle activity reduction performance, the BO algorithm is more sufficient during each iteration compared to CMA-ES and takes fewer iterations to converge. For each iteration, the sEMG signal of the RF muscle was able to update and reflect the influence of the current assistive torque pattern within 25 s, which is faster than the method with metabolic expenditure measurement (more than 120 s (Zhang et al., 2017; Ding et al., 2018). The results of optimized torque patterns’ verification experiments show that the sEMG-based HIL optimization is a promising solution for exoskeleton-assisted walking control to reduce muscle activities.

### 4.2 Subject experiences

In the HIL optimization stage, a wide range (with an amplitude between 0 Nm and 10 Nm) of torque profiles is applied and tuned through the HIL optimization. Most subjects felt that neither high nor low torque amplitude was comfortable during exoskeleton-assisted walking. The high torque assistance would cause human-exoskeleton joint misalignment, which may lead to resistance of the participants’ hip joint to the assist torque. With low torque assistance, the subject felt more restraint and weight burden from the hip exoskeleton. Consequently, the optimized torque patterns that are all within acceptable torque amplitudes are reasonable. Another common experience from all participants is that the timing of peak torque influences the feeling of assistance most. Meanwhile, the smoothness of the torque profile can also affect comfort. Both Subject 2 and Subject 4 pointed out that PA mode was more comfortable than CA and BA modes because there were transient jumps of assist torque during HIL optimization, which felt like a sudden drag on the thigh rather than a smooth push and pull of the thigh for walking assistance. This could be optimized in assistive torque profile parameterization by ensuring the continuation of the first derivative of the torque profile.

### 4.3 Study limitations

It is worth noting that this study has limitations. One is that only the RF muscle activity was measured for HIL optimization of torque patterns. Although the literature has shown that the RF muscle activity was reduced most when assisted by the hip exoskeleton compared to the unpowered condition (Young et al., 2017a), it would be valuable to select a cost function that covers muscle activities of all hip joint-related muscles. Another limitation is that only a single walking scenario, i.e., treadmill walking with constant speed, was tested. In daily life, humans do not solely locomote on level ground and constantly adjust their gait activities and walking speeds. Further experiments under various scenarios should be conducted to systematically study the best assistive torque patterns. In addition, only four subjects were recruited for the trials. Having more participants would better strengthen our results.

## 5 Conclusion

In conclusion, we developed a portable hip exoskeleton with compatible joint alignment mechanisms. The sEMG-based HIL optimization of torque patterns was presented, and the RF muscle activity was evaluated to validate the effect of the exoskeleton. The key findings of this study showed a decrease in RF muscle activity when the sEMG-based HIL optimization was applied to assist torque generation. Furthermore, by applying muscle activities to the upper-layer control loop, this study preliminarily revealed the human body’s muscle adaptation mechanisms associated with the assist torque patterns, which are still an open challenge in the field of wearable robots. Future work will focus on exploring the characterization of muscle fatigue and muscle synergy during wearable robot-assisted walking and developing a new HIL optimization for effective and natural human-robot interaction.

## Data Availability

The raw data supporting the conclusion of this article will be made available by the authors, without undue reservation.
